# Supplementary material for: From sequence to enzyme mechanism using multi-label machine learning
Source: BMC Bioinformatics. 2014 May 19;15:150. doi: 10.1186/1471-2105-15-150 (PMC4229970; doi:10.1186/1471-2105-15-150)
Supplement: Additional file 2 — Java code of ml2db. Additional file ml2db_code.tar.gz contains the Java source code to run the multi-label machine learning experiments and save the results to database. The code’s Javadoc is included. [file 1471-2105-15-150-S2.zip › additional file 2/ml2db/ml2db/doc/mulan/classifier/meta/EnsembleOfSubsetLearners.html]

EnsembleOfSubsetLearners


JavaScript is disabled on your browser.


- Overview
- Package
- Class
- Use
- Tree
- Deprecated
- Index
- Help

- Prev Class
- Next Class

- Frames
- No Frames

- All Classes

- Summary:
- Nested |
- Field |
- Constr |
- Method

- Detail:
- Field |
- Constr |
- Method


mulan.classifier.meta

## Class EnsembleOfSubsetLearners

- java.lang.Object
- - mulan.classifier.MultiLabelLearnerBase
  - - mulan.classifier.meta.MultiLabelMetaLearner
    - - mulan.classifier.meta.EnsembleOfSubsetLearners

- All Implemented Interfaces:
  :   java.io.Serializable, MultiLabelLearner, weka.core.TechnicalInformationHandler

  ---

    

  ```
  public class EnsembleOfSubsetLearners
  extends MultiLabelMetaLearner
  ```

  A class for gathering several different SubsetLearners into a composite ensemble model. <br> <br> The label set partitions for participation in ensemble are selected using their dependence weight from the large number of randomly generated possible partitions. The type of the learned dependencies is determined by the {@link mulan.data.LabelPairsDependenceIdentifier} supplied to the class constructor. Two strategies for selecting ensemble partitions exists: (1) to select the highly weighted ones and (2) to select most different from the highly weighted ones. The strategy to be used is determined by the {@link #selectDiverseModels} parameter which is 'true' by default.

  Version:
  :   30.11.2010

  Author:
  :   Lena Chekina (lenat@bgu.ac.il)

  See Also:
  :   Serialized Form

- - ### Constructor Summary

    Constructors

    Constructor and Description

    `EnsembleOfSubsetLearners()`

    Default constructor.

    `EnsembleOfSubsetLearners(MultiLabelLearner aMultiLabelLearner,
    weka.classifiers.Classifier aSingleLabelLearner,
    LabelPairsDependenceIdentifier aDependenceIdentifier,
    int models)`

    Initialize EnsembleOfSubset with multilabel and single label learners, a
    method for labels dependence identification and number of models to
    constitute the ensemble.
  - ### Method Summary

    Methods

    | Modifier and Type | Method and Description |
    | --- | --- |
    | `java.util.List<int[][]>` | `createRandomSets(int numLabels, int numSets)` Creates the specified number of randomly generated possible label set partitions consisting of the specified number of labels.. |
    | `int` | `getNumModels()` |
    | `weka.core.TechnicalInformation` | `getTechnicalInformation()` Returns an instance of a TechnicalInformation object, containing detailed information about the technical background of this class, e.g., paper reference or book this class is based on. |
    | `java.lang.String` | `globalInfo()` Returns a string describing the multi-label learner. |
    | `boolean` | `isSelectDiverseModels()` |
    | `static java.lang.String` | `partitionToString(int[][] partition)` Returns a string representation of the labels partition. |
    | `void` | `setDependenceIdentifier(LabelPairsDependenceIdentifier dependenceIdentifier)` |
    | `static void` | `setDynamicDiversityThreshold(double dynamicDiversityThreshold)` |
    | `void` | `setNumModels(int models)` |
    | `static void` | `setNumOfPartitionsForDiversity(int numOfPartitionsForDiversity)` |
    | `static void` | `setNumOfRandomPartitions(int numOfRandomPartitions)` |
    | `void` | `setRnd(java.util.Random rnd)` |
    | `void` | `setSeed(int x)` |
    | `void` | `setSelectDiverseModels(boolean selectDiverseModels)` |
    | `void` | `setThreshold(double threshold)` |
    | `void` | `setUseSubsetLearnerCache(boolean useSubsetcache)` |

    - ### Methods inherited from class mulan.classifier.meta.MultiLabelMetaLearner

      `getBaseLearner`
    - ### Methods inherited from class mulan.classifier.MultiLabelLearnerBase

      `build, getDebug, isUpdatable, makeCopy, makePrediction, setDebug`
    - ### Methods inherited from class java.lang.Object

      `equals, getClass, hashCode, notify, notifyAll, toString, wait, wait, wait`

- - ### Constructor Detail


    - #### EnsembleOfSubsetLearners

      ```
      public EnsembleOfSubsetLearners()
      ```

      Default constructor. Can be used for accessing class utility methods.


    - #### EnsembleOfSubsetLearners

      ```
      public EnsembleOfSubsetLearners(MultiLabelLearner aMultiLabelLearner,
                              weka.classifiers.Classifier aSingleLabelLearner,
                              LabelPairsDependenceIdentifier aDependenceIdentifier,
                              int models)
      ```

      Initialize EnsembleOfSubset with multilabel and single label learners, a
      method for labels dependence identification and number of models to
      constitute the ensemble.

      Parameters:
      :   `aMultiLabelLearner` - the learner for multilabel classification
      :   `aSingleLabelLearner` - the learner for single label classification
      :   `aDependenceIdentifier` - the method for label pairs dependence
          identification
      :   `models` - the number of models
  - ### Method Detail


    - #### getTechnicalInformation

      ```
      public weka.core.TechnicalInformation getTechnicalInformation()
      ```

      **Description copied from class: `MultiLabelLearnerBase`**

      Returns an instance of a TechnicalInformation object, containing detailed
      information about the technical background of this class, e.g., paper
      reference or book this class is based on.

      **Specified by:**
      :   `getTechnicalInformation` in interface `weka.core.TechnicalInformationHandler`

      **Specified by:**
      :   `getTechnicalInformation` in class `MultiLabelLearnerBase`

      Returns:
      :   the technical information about this class


    - #### createRandomSets

      ```
      public java.util.List<int[][]> createRandomSets(int numLabels,
                                             int numSets)
      ```

      Creates the specified number of randomly generated possible label set
      partitions consisting of the specified number of labels..

      Parameters:
      :   `numLabels` - - number of labels
      :   `numSets` - - number of random partitions to generate

      Returns:
      :   a list of generated partitions


    - #### partitionToString

      ```
      public static java.lang.String partitionToString(int[][] partition)
      ```

      Returns a string representation of the labels partition.

      Parameters:
      :   `partition` - - a label set partition

      Returns:
      :   a string representation of the labels partition


    - #### setRnd

      ```
      public void setRnd(java.util.Random rnd)
      ```

      Parameters:
      :   `rnd` -


    - #### setThreshold

      ```
      public void setThreshold(double threshold)
      ```

      Parameters:
      :   `threshold` -


    - #### setDependenceIdentifier

      ```
      public void setDependenceIdentifier(LabelPairsDependenceIdentifier dependenceIdentifier)
      ```

      Parameters:
      :   `dependenceIdentifier` -


    - #### setSeed

      ```
      public void setSeed(int x)
      ```

      Parameters:
      :   `x` -


    - #### setNumModels

      ```
      public void setNumModels(int models)
      ```

      Parameters:
      :   `models` -


    - #### getNumModels

      ```
      public int getNumModels()
      ```

      Returns:


    - #### isSelectDiverseModels

      ```
      public boolean isSelectDiverseModels()
      ```

      Returns:


    - #### setSelectDiverseModels

      ```
      public void setSelectDiverseModels(boolean selectDiverseModels)
      ```

      Parameters:
      :   `selectDiverseModels` -


    - #### setUseSubsetLearnerCache

      ```
      public void setUseSubsetLearnerCache(boolean useSubsetcache)
      ```

      Parameters:
      :   `useSubsetcache` -


    - #### setNumOfRandomPartitions

      ```
      public static void setNumOfRandomPartitions(int numOfRandomPartitions)
      ```

      Parameters:
      :   `numOfRandomPartitions` -


    - #### setNumOfPartitionsForDiversity

      ```
      public static void setNumOfPartitionsForDiversity(int numOfPartitionsForDiversity)
      ```

      Parameters:
      :   `numOfPartitionsForDiversity` -


    - #### setDynamicDiversityThreshold

      ```
      public static void setDynamicDiversityThreshold(double dynamicDiversityThreshold)
      ```

      Parameters:
      :   `dynamicDiversityThreshold` -


    - #### globalInfo

      ```
      public java.lang.String globalInfo()
      ```

      **Description copied from class: `MultiLabelLearnerBase`**

      Returns a string describing the multi-label learner.

      **Specified by:**
      :   `globalInfo` in class `MultiLabelLearnerBase`

      Returns:
      :   a description suitable for displaying in a future gui


- Overview
- Package
- Class
- Use
- Tree
- Deprecated
- Index
- Help

- Prev Class
- Next Class

- Frames
- No Frames

- All Classes

- Summary:
- Nested |
- Field |
- Constr |
- Method

- Detail:
- Field |
- Constr |
- Method
